# Epigenomic Analysis of RAD51 ChIP-seq Data Reveals *cis*-regulatory Elements Associated with Autophagy in Cancer Cell Lines

**DOI:** 10.3390/cancers13112547

**Published:** 2021-05-22

**Authors:** Keunsoo Kang, Yoonjung Choi, Hyeonjin Moon, Chaelin You, Minjin Seo, Geunho Kwon, Jahyun Yun, Boram Beck, Kyuho Kang

**Affiliations:** 1 Department of Microbiology, College of Science & Technology, Dankook University, Cheonan 31116, Korea; moonhj0821@naver.com (H.M.); happiness21_@naver.com (M.S.); wkgusthfk12@naver.com (J.Y.); 2 Deargen Inc., 193, Munji-ro, Yuseong-gu, Daejeon 34051, Korea; yoonjungc@deargen.me (Y.C.); brbr777@deargen.me (B.B.); 3 Department of Biological Sciences and Biotechnology, Chungbuk National University, Cheongju 28644, Korea; clyu104@cbnu.ac.kr (C.Y.); geunho93@cbnu.ac.kr (G.K.)

**Keywords:** RAD51, autophagy, *cis*-regulatory element, E-box, USF1, USF2, ChIP-seq, gene regulation

## Abstract

**Simple Summary:**

RAD51 is a key enzyme involved in homologous recombination during DNA double-strand break repair. However, recent studies suggest that non-canonical roles of RAD51 may exist. The aim of our study was to assess regulatory roles of RAD51 by reanalyzing RAD51 ChIP-seq data in GM12878, HepG2, K562, and MCF-7 cell lines. We identified 5137, 2611, 7192, and 3498 RAD51-associated *cis*-regulatory elements in GM12878, HepG2, K562, and MCF-7 cell lines, respectively. Intriguingly, gene ontology analysis revealed that promoters of the autophagy pathway-related genes were most significantly occupied by RAD51 in all four cell lines, predicting a non-canonical role of RAD51 in regulating autophagy-related genes.

**Abstract:**

RAD51 is a recombinase that plays a pivotal role in homologous recombination. Although the role of RAD51 in homologous recombination has been extensively studied, it is unclear whether RAD51 can be involved in gene regulation as a co-factor. In this study, we found evidence that RAD51 may contribute to the regulation of genes involved in the autophagy pathway with E-box proteins such as USF1, USF2, and/or MITF in GM12878, HepG2, K562, and MCF-7 cell lines. The canonical USF binding motif (CACGTG) was significantly identified at RAD51-bound *cis*-regulatory elements in all four cell lines. In addition, genome-wide USF1, USF2, and/or MITF-binding regions significantly coincided with the RAD51-associated *cis*-regulatory elements in the same cell line. Interestingly, the promoters of genes associated with the autophagy pathway, such as ATG3 and ATG5, were significantly occupied by RAD51 and regulated by RAD51 in HepG2 and MCF-7 cell lines. Taken together, these results unveiled a novel role of RAD51 and provided evidence that RAD51-associated *cis*-regulatory elements could possibly be involved in regulating autophagy-related genes with E-box binding proteins.

## 1. Introduction

RAD51 plays a crucial role in homologous recombination (HR) during DNA double-strand break (DSB) repair [1,2,3,4]. RAD51 catalyzes the homology search and ATP-dependent DNA strand exchange of the bound single DNA strand with the complementary strand within the duplex. In addition to its well-known role as a recombinase in DSB repair, it has also been studied in a non-enzymatic role in multiple processes in response to replication stress [5]. RAD51 promotes the reversal of the replication fork. It also protects nascent DNA at regressed forks from degradation. Recently, a new role for the immune response of RAD51 has been suggested [6]. Inhibition of RAD51 results in the accumulation of self-DNA in the cytosol, which in turn increases the STING-dependent innate immune response. Since these various roles of RAD51 in DNA damage are closely related to genome stability, the normal cell cycle, and immunity, RAD51 has been considered a promising therapeutic target for various cancers including lung, breast, melanoma, and pancreatic cancer [7,8,9,10,11].

RAD51 is highly expressed in various cancers including breast, lung, and pancreatic cancers [12,13,14]. It is also known to interact directly with tumor suppressor proteins, such as BRCA1 and BRCA2, which play important roles in DNA repair in response to damage [15]. Indeed, BRCA2 is known to control the DNA-binding ability and intracellular localization of RAD51 [11]. Overexpressed RAD51 causes increased survival of tumor cells and resistance to DNA-damaging treatments such as radiotherapy and chemotherapy [16]. The hyperactivation of RAD51 contributes to increased homologous recombination associated with tumor progression and metastasis [8]. For example, in ER-positive breast cancer, elevated RAD51 expression is related to resistance to neoadjuvant endocrine therapy including aromatase inhibitor, and the poor survival of patients [17]. Intriguingly, this role of RAD51 is also related to the function of autophagy in cancers. Although autophagy seems to have a tumor-suppressive role in normal cells, it provides resistance to DNA repair targeted cancer therapy through the inhibition of genomic instability [18,19]. Mechanistically, a loss of autophagy leads to elevated p62, the ubiquitin-binding protein. Accumulated nuclear p62 suppresses HR-associated DSB repair through the proteasomal degradation of RAD51 and FLNA [20,21]. In protein–protein interactions, one study suggested that the relationship between RAD51 and autophagy is associated with checkpoint kinase 1 (CHK1) in an esophageal cancer model by inhibiting autophagy [22]. Thus, in several cancers, the inhibition of autophagy promotes DNA damage and a susceptibility to cancer treatment by the downregulation of RAD51 [23,24,25]. On the contrary, one study reported that inducing autophagy reduces RAD51 expression in non-small cell lung cancer (NSCLC) cell models [26]. Taken together with the case studies above, the RAD51–autophagy interaction seems to have the nature of a two-faced role of autophagy in relation to cancers [27], where a different point of view may be required to understand the RAD51–autophage axis. In short, it has been found that autophagy regulates RAD51 protein, but the precise molecular mechanisms underlying how RAD51 regulates autophagy in cancers are still elusive.

Two recent investigations into the molecular mechanism of RAD51 have been extended to the transcriptional level through a genome-wide approach, using chromatin immunoprecipitation followed by sequencing (ChIP-seq). First, RAD51 was recruited to the active chromatin regions, which are preferentially repaired by HR [28]. These sites show the transcriptional elongation-associated histone mark, histone 3 trimethylated lysine 36 (H3K36me3). Second, the genome-wide mapping of DSBs shows that the co-occupancy of RAD51, together with the transcription factor, TEAD4, at oncogenic super-enhancers is associated with the overexpression of oncogenes [29]. Despite these studies, the exact mechanisms by which RAD51 regulates gene expression are not fully understood. 

In this study, we investigated the novel role of RAD51 in regulating genes at the transcriptional level, by reanalyzing RAD51 ChIP-seq data in multiple cancer cell lines, such as GM12878, HepG2, K562, and MCF-7. We found that genome-wide RAD51 bindings were more strongly enriched at the active promoters with upstream stimulating factor (USF1 and USF2) motifs. Deeper analysis revealed that RAD51-associated *cis*-regulatory elements coincided with E-box proteins such as USF1, USF2, and/or MITF, especially on the genes associated with autophagy and lysosome. Our findings demonstrated an unrecognized function of RAD51 in transcriptional regulation of genes, which is related to autophagy in cancers.

## 2. Materials and Methods

### 2.1. ChIP-seq Data Analysis

ChIP-seq data were downloaded from the ENCODE database (https://www.encodeproject.org/, accessed on 12 March 2021) [30]. The accession numbers for all samples can be found in Table S1. Raw files (FASTQ) were trimmed by quality, using Trim Galore (https://www.bioinformatics.babraham.ac.uk/projects/trim_galore/, accessed on 12 March 2021, version 0.6.4) with default parameters. Trimmed reads were aligned to the human genome (hg38 genome assembly) using Bowtie2 (version 2.3.4.1) with default parameters [31]. Duplicates of mapped reads were removed using Sambamba (version 0.6.7) with default parameters [32]. Peaks were identified using HOMER (version 4.11.1; findPeaks) [33] with the corresponding control sample (Table S1). A false discovery rate (FDR)-adjusted *p*-value cutoff of 0.001 was used for the analysis. To further identify reliable peaks, those peaks that did not overlap at least 50% of regions between the biological replicates were discarded, using Bedtools (version 2.26.0) [34].

### 2.2. DNA Binding Motif Analysis

Motifs, which were significantly associated with given binding sites (150 bp), were identified using HOMER (findMotifsGenome.pl script) with the following parameters: -size 150 -mask.

### 2.3. Gene Ontology Analysis

Gene ontology (GO) analysis was conducted using Metascape (http://metascape.org/, accessed on 12 March 2021) [35] with the genes closest to the top 500 peaks (descending order of RPKM values in Table S3) in each cell line.

### 2.4. Data Visualization

A Venn diagram was generated using InteractiVenn (http://www.interactivenn.net/, accessed on 12 March 2021) [36], and snapshots of gene loci were obtained from the IGV application (https://igv.org/app/, accessed on 12 March 2021) [37]. Heatmaps were generated using Deeptools (version 3.2.1) [38] based on peaks with read per million (RPM)-normalized enrichment signal files (bigwig) of samples.

### 2.5. Kaplan–Meier Survival Analysis

Kaplan–Meier (KM) survival analysis was performed using Oncolnc (http://www.oncolnc.org/, accessed on 12 March 2021) [39]. Patients were divided into high (50%) or low (50%) groups based on the expression level of the *RAD51* gene.

### 2.6. Cell Culture and Reagents

The human breast cancer cell line MCF7 and the human liver cancer cell line HepG2 were obtained from the Korean cell line bank (KCLB). MCF7 cells were cultured in RPMI 1640, and HepG2 cells were cultured in minimum essential medium (MEM) (Gipco, Waltham, MA. USA). All media were supplemented with 10% fetal bovine serum (FBS; Gibco) and 1% antibiotic–antimycotic (Gibco). All cell lines were routinely cultured in a humidified 5% CO2 incubator at 37 °C. MCF7 and HepG2 cells were seeded onto 12-well plates and incubated with cisplatin (Sigma-Aldrich, St. Louis, MO. USA) at various concentrations (0, 2, 4, 6, and 8 μg/mL) and RS-1 (medchemexpress) at various concentrations (0, 5, and 10 μM) and B02 (medchemexpress) at various concentrations (0, 5, 10, and 15 μM) for 24 h at 37 °C in an atmosphere containing 5% CO2.

### 2.7. Real-Time Quantitative PCR

Total RNA was extracted from cells using Ribospin II (GeneAll), and total RNA was reverse transcribed using the RevertAid First Strand cDNA Synthesis kit (Thermo Fisher Scientific, Waltham, MA. USA). Real-time PCR was performed in replicate with TOPreal qPCR 2X PreMIX (SYBR Green with low ROX). Rotor-Gene Q software 2.3.1 was used for capturing and analyzing spectral data. Relative mRNA expression of *ATG3* and *ATG5* was normalized to the house-keeping gene TBP. Primers for these genes are indicated in Table S2.

### 2.8. Statistical Analysis

All quantitative data are presented as the mean ± SEM. One-way analysis of variance (ANOVA) was used to identify statistically significant data. *p*-values < 0.05 were considered significant. Statistical analyses were performed using GraphPad Prism 8 (GraphPad Software, Inc.; http://www.graphpad.com/scientific-software/prism/, accessed on 12 March 2021).

## 3. Results

### 3.1. Analysis of Genome-Wide RAD51 Binding Sites in GM12878, HepG2, K562, and MCF-7 Cell Lines

**Figure 1:**
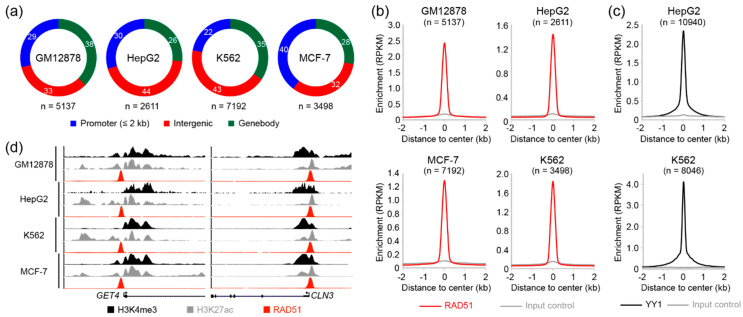
Characteristics of genome-wide RAD51 binding sites. (**a**) Pie charts show the percentage of genome-wide RAD51 binding sites according to gene annotation. Line plots show average enrichment of RAD51 binding at RAD51 binding sites (**b**) and YY1 binding at YY1 binding sites (**c**). (**d**) Enrichment of active (H3K4me3 and H3K27ac) histone modifications around RAD51 binding sites near the *GET4* and *CLN3* genes is shown.

Although RAD51 plays a central role in HR, recent studies using chromatin immunoprecipitation followed by sequencing (ChIP-seq) revealed that RAD51 binds to transcriptionally active sites [28,29], suggesting a potential role for RAD51 in gene regulation. To investigate a novel role of RAD51 in regulating genes, we reanalyzed RAD51 ChIP-seq data deposited in the encyclopedia of DNA elements (ENCODE) database (https://www.encodeproject.org/, accessed on 12 March 2021) [30]. There were four RAD51 ChIP-seq datasets available, which were performed in GM12878, HepG2, K562, and MCF-7 cell lines. The accession number of all samples used in this study can be found in Table S1. Two biological replicates of RAD51 and the corresponding control ChIP-seq performed on GM12878, HepG2, K562, and MCF-7 cell lines were analyzed as described previously [40]. RAD51 binding sites (peaks) in each cell line were identified using HOMER with a false discovery rate (FDR)-adjusted *p*-value cutoff of 0.001. To further filter out non-reliable RAD51 binding sites, we discarded the peaks that did not overlap between biological replicates. The analysis revealed that the characteristic of genome-wide RAD51 binding was surprisingly similar to those of typical transcription factors. For example, there were totals of 5137, 2611, 7192, and 3498 RAD51-associated *cis*-regulatory elements in GM12878, HepG2, K562, and MCF-7 cell lines, respectively (Table S3), and up to 44% and 40% of RAD51 binding sites were located in the intergenic and promoter regions, respectively (Figure 1a). This result show that the majority of genome-wide RAD51 binding sites coincided with promoters, which are the most important regulatory elements for gene regulation, suggesting that RAD51 may be involved in gene regulatory mechanisms. In addition, the average enrichment of RAD51 to their binding sites clearly showed a sharp peak-like shape at the center of the peaks (Figure 1b), which is typically observed in those of transcription factors, as shown, for example, in the YY1 transcription factor (Figure 1c). Manual investigation of RAD51 binding sites also indicated that RAD51 could participate in regulating nearby genes. For instance, the promoter regions of the GET4 and CLN3 genes were significantly occupied by RAD51 in the cells of all four cell lines (Figure 1d). Accordingly, active histone modifications, such as H3K4me3 and H3K27ac, were markedly enriched around the binding sites. To further gain biological insights into RAD51, regarding gene regulation, the RAD51 binding sites were categorized into two groups (promoter-associated or enhancer-associated), depending on the distance to the transcription start sites (TSSs) of known genes. The promoter-associated RAD51 group was defined as RAD51 binding sites within the 2 kb flanking regions of the TSSs, while the enhancer-associated RAD51 group contained the rest of the RAD51 binding sites that did not belong to the promoter-associated group. Intriguingly, promoter-associated RAD51 peaks showed stronger RAD51 enrichment than enhancer-associated RAD51 binding sites, and expression levels of the genes with the promoter-associated RAD51 binding sites were significantly higher than those with the enhancer-associated RAD51 peaks (Figure S1). Collectively, these results provided evidence that RAD51 may be involved in gene regulation, possibly through the mechanism similar to those of transcription factors. This unexpected role of RAD51 has not been addressed previously, due to the well-known enzymatic role of RAD51 in homologous recombination.

### 3.2. DNA Binding Motif Analysis of RAD51 Binding Sites in GM12878, HepG2, K562, and MCF-7 Cell Lines

**Figure 2:**
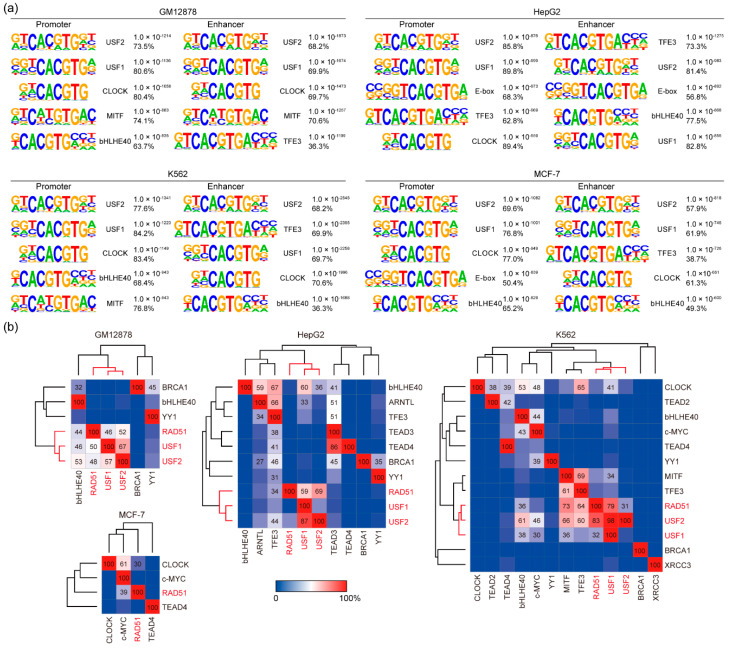
Motif analysis of genome-wide RAD51 binding sites in GM12878, HepG2, K562, and MCF-7 cell lines. (**a**) The top five motifs identified in each set of RAD51 binding sites are shown. (**b**) Heatmaps show the percentage of co-occupancy between given proteins. The percentage was calculated by dividing the number of co-occupied sites by the total number of binding sites on a given protein (row). Hierarchical clustering based on the co-occupancy percentage was conducted using the average-linkage method with the one-minus Pearson correlation metric.

To discover DNA binding motifs recognized preferentially by RAD51, motif analysis was performed on the RAD51 peaks identified in GM12878, HepG2, K562, and MCF-7 cell lines. HOMER was used to assess overrepresented motifs in the flanking (150 bp) regions relative to the center of the RAD51 peaks. Surprisingly, the known binding motifs for the upstream stimulating factors (USF1 and USF2), followed by CLOCK, MITF, bHLHE40, and TFE3, were significantly overrepresented at the RAD51 binding sites in all four cell lines (Figure 2a). For example, a significant presence of the USF2 motif was found in RAD51 binding sites (73.5%) in GM12878 cells with a *p*-value of 1.0 × 10^−1214^. Notably, all of these transcription factors are known to bind to a consensus DNA element called an enhancer box (E-box), which harbors the palindromic DNA sequence “CACGTG”, and these E-box motifs were highly specific for RAD51 binding since motifs found at H3K4me3-enriched regions (active histone modification) in the same cell line were much less significant than the E-box motifs in terms of the *p*-value (Figure S2). In addition, there is no substantial difference in these motifs between the promoter and enhancer regions in all four cell lines (Figure 2a). Accordingly, these results led us to investigate a novel role for RAD51 in gene regulation besides the well-known enzymatic role in homologous recombination. Based on the above results (Figure 1 and Figure 2), we hypothesized that RAD51 could participate in gene regulation by binding to the target site with E-box binding proteins. To verify the hypothesis, we first reanalyzed available USF1, USF2, CLOCK, MITF, bHLHE40, and/or TFE3 ChIP-seq data in the ENCODE database to pinpoint which E-box binding transcription factors predominantly coincided with the RAD51 binding sites. Unfortunately, some of the E-box binding protein ChIP-seq data were not available. Therefore, only those available ChIP-seq data were reanalyzed for the analysis. The results showed that most RAD51 binding sites coincided with USF1 and/or USF2 in GM12878, HepG2, and K562 cell lines (Figure 2b). For instance, 52%, 69%, and 31% of RAD51 binding sites in GM12878, HepG2, and K562 cell lines, respectively, overlapped with USF2 peaks. Reciprocally, 48%, 29%, and 83% of USF2 binding sites in GM12878, HepG2, and K562 cell lines, respectively, coincided with RAD51 peaks. Almost similar results were obtained between RAD51 and USF1. Hierarchical clustering, based on the percentage of overlaps between the given proteins, indicated that RAD51, USF1, and USF2 were well-clustered among all E-box proteins reanalyzed in this study. This is an unexpected result because most, if not all, RAD51 studies never reported the co-localization of RAD51 and USF factors. To ascertain the finding, enrichment heatmaps were drawn, based on the binding sites of each protein, along with all available samples (Figure S3). The results confirmed that the RAD51 binding sites mostly coincided with USF1 and USF2 binding sites, followed by MITF and TFE3 peaks. In contrast, the binding sites of proteins known to be associated with RAD51 during homologous recombination, such as BRCA1, TEAD3, and TEAD4, rarely overlapped with the RAD51 binding sites. Overall, these results indicated that RAD51 could be involved in gene regulation with E-box binding proteins such as USF1, USF2, and/or MITF, which has not been addressed previously.

### 3.3. RAD51 and Autophagy

**Figure 3:**
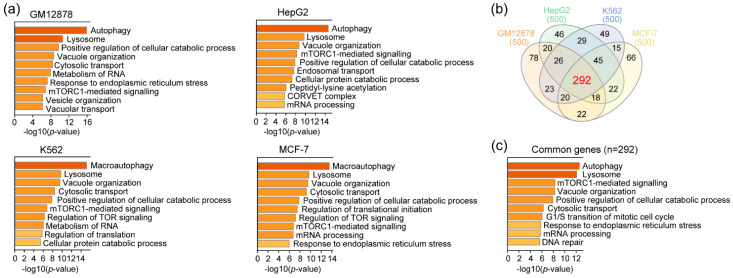
Predicted biological pathways associated with the top 500 RAD51 binding sites. (**a**) Gene ontology analysis was performed using Metascape [34] with the nearest genes of the top 500 RAD51 binding sites. (**b**) The Venn diagram shows the number of overlapping RAD51 target genes between cell lines. (**c**) Biological pathways that were related to common RAD51 target genes were predicted.

**Figure 4:**
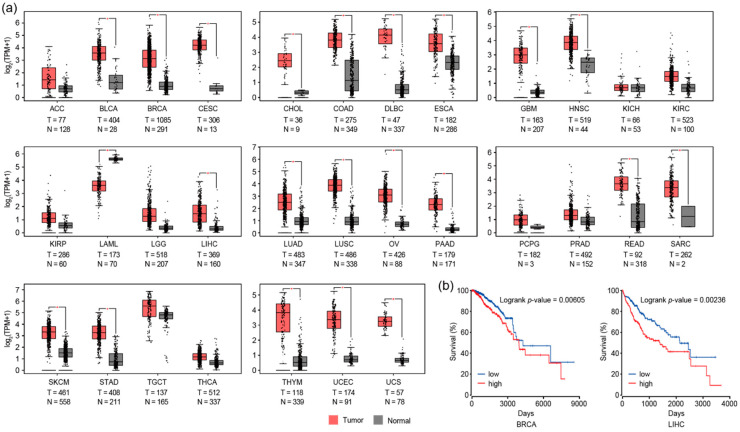
Expression levels of the RAD51 gene in various types of cancer. (**a**) Box plots show normalized expression levels (transcripts per kilobase million, TPM) of the RAD51 gene in tumor tissues and corresponding normal controls. *p-value < 0.01. (**b**) The prognosis of patients diagnosed with BRCA or LIHC was estimated and plotted in Kaplan–Meier survival plots. The patients were divided into two groups (high and low) based on the expression level of the RAD51 gene.

Our comprehensive analysis of genome-wide RAD51 binding sites revealed that those in GM12878, HepG2, and K562 cells significantly coincided with E-box binding proteins such as USF1 and USF2 (Figure 2b). The motif analysis (Figure 2a) also indicated that USF1 and USF2 motifs occurred significantly in those RAD51 binding sites. Thus, these results collectively indicated that RAD51 might be involved in gene regulation by binding indirectly to regulatory elements in the genome via E-box binding proteins. To assess the potential biological pathway that the RAD51-mediated gene regulation could contribute, gene ontology (GO) analysis was performed with the genes whose promoters were bound by the top 500 RAD51 peaks (in descending order of normalized mapped reads) (Table S3). Previously, this in silico analysis strategy successfully identified the known FOXM1-mediated pathway, such as the cell cycle, in various cancer cell lines [40]. The result showed that autophagy and lysosome pathways were the main two pathways significantly associated with the top 500 RAD51 binding sites in all four cancer cell lines (Figure 3a). This is an unexpected finding, predicting a gene regulatory role for RAD51 in the autophagy pathway, as most RAD51 studies, to the best of our knowledge, have focused on the enzymatic role of RAD51 in homologous recombination. These cancer cell lines are all derived from different types of cancer. Nevertheless, the genes related to the autophagy pathway were commonly identified in all four cell lines as RAD51 target genes. Therefore, this finding suggests that the role of RAD51 in regulating the autophagy pathway might be common in at least some cancer types. To further identify a core gene set, genes associated with the top 500 RAD51 binding sites in all four cell lines were intersected (Figure 3b). As expected, three-fifths of the RAD51 target genes (292 genes) were shared in GM12878, HepG2, K562, and MCF-7 cell lines (Table S4). Gene ontology analysis of the core gene set verified that the autophagy pathway was the pathway most significantly associated with these common genes (Figure 3c). Next, we determined whether the expression level of RAD51 was indeed associated with clinical features of various cancer patients. Intriguingly, the expression of the RAD51 gene was significantly upregulated in most cancers, as compared to corresponding normal controls (Figure 4a). For example, among 31 types of cancer, RAD51 was significantly overexpressed in 21 cancers. In contrast, its expression was significantly reduced only in acute myeloid leukemia (LAML). Expression levels of the RAD51 gene were also significantly associated with the prognosis of patients in some cancers. In breast invasive carcinoma (BRCA) and liver hepatocellular carcinoma (LIHC), patients showing higher expression of the RAD51 gene showed significantly poor prognoses (Figure 4b), as compared to the other group. Collectively, these results suggested that RAD51 may contribute to the regulation of the autophagy pathway in GM12878, HepG2, K562, and MCF-7 cells, and the expression level of the RAD51 gene could at least be used to predict outcomes for breast and liver cancer patients. 

**Figure 5:**
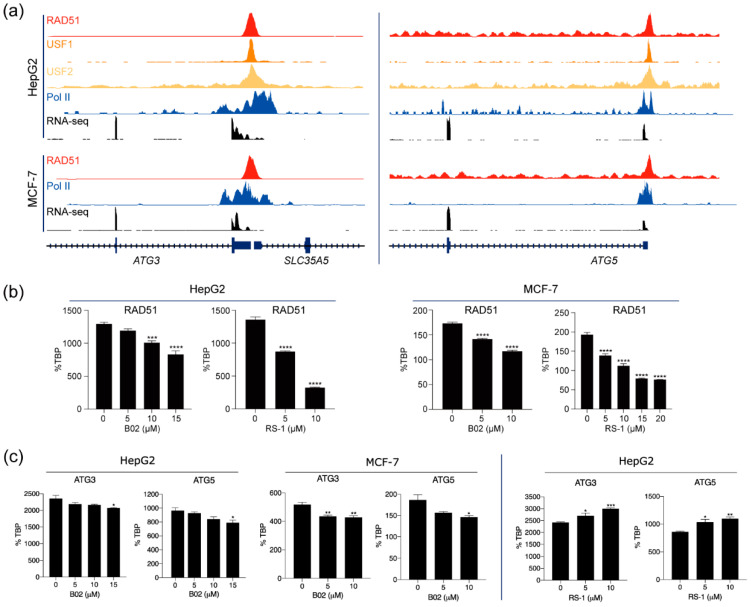
RAD51 is involved in the regulation of autophagy-associated genes in HepG2 and MCF7 cell lines. (**a**) Snapshots of ChIP-seq browser tracks for the ATG3 and ATG5 genes are shown. (**b**) Transcript levels of RAD51 were assessed in HepG2 and MCF-7 cells treated with B02 or RS-1 at different concentrations for 24 h. (**c**) Transcript levels of ATG3 and ATG5 were assessed in HepG2 and MCF7 cells treated with B02 (left) or RS-1 (right) at different concentrations for 24 h. TBP was used for the normalization of each cell line level. * *p* < 0.05; ** *p* < 0.01; *** *p* < 0.001; **** *p* < 0.0001. The analysis was performed using one-way ANOVA. Data are shown as mean ± SEM.

To further validate the predicted role of RAD51 in regulating autophagy-related pathways in vitro, we first checked expression levels of the RAD51 gene in various cancer cell lines (Figure S4). As expected, the RAD51 gene is highly expressed in most cancer cell lines. Next, we selected two key genes, ATG3 and ATG5, which are involved in the autophagy pathways, to test whether RAD51 could regulate the expression of those genes. These genes were selected since their promoters were strongly occupied by RAD51, RNA polymerase II, and/or USF1 and USF2 in the HepG2 and MCF-7 cell lines, and therefore they were highly expressed in these cell lines, as confirmed by RNA-seq (Figure 5a). To assess a novel regulatory role of RAD51 for these genes, we first investigated whether a reduction in RAD51 transcripts altered the expression levels of the ATG3 and ATG5 genes in the HepG2 and MCF-7 cell lines, using the small interfering RNA (siRNA) technique. Although the expression of RAD51 was significantly reduced in both cell lines, there were no significant changes in the expression levels of the ATG3 and ATG5 genes (Figure S5). This result suggests that the nuclear RAD51 protein may remain. Therefore, two compounds, a small molecule RAD51 inhibitor (B02) and a RAD51-stimulatory compound (RS-1), were used to disrupt or promote direct binding of RAD51 to DNA regulatory elements. When HepG2 and MCF-7 cells were treated with B02, the expression level of ATG3 and ATG5 genes was significantly reduced in a dose-dependent manner (Figure 5c). Conversely, the expression levels of ATG3 and ATG5 genes increased when treated with RS-1 in HepG2 cells (Figure 5c). These results suggest that RAD51 contributes to the regulation of autophagy-related genes, such as ATG3 and ATG5, in HepG2 and MCF-7 cells, in a DNA-binding-dependent manner. Further mechanistic studies are needed to fully support this hypothesis.

## 4. Discussion

Since RAD51 plays a pivotal role in homologous recombination, almost all studies regarding RAD51 have focused primarily on the role of RAD51 in homologous recombination. This is because RAD51 is a recombinase that filaments on double- and single-stranded DNA [41], in order to mediate strand exchange during homologous recombination [42,43]. In this study, we provide some evidence that RAD51 may be involved in regulating genes related to the autophagy pathway, in cooperation with E-box binding proteins such as USF1 and USF2. First, RAD51 was found to bind to up to several thousand target sites on the genome in GM12878, HepG2, K562, and MCF-7 cells (Figure 1). Motif analysis based on these RAD51 binding sites in the four cancer cell lines indicated that RAD51 binding did not occur on random sites of the genome, but did bind significantly on regulatory elements with the cognate E-box motif (CACGTG) in all four cell lines (Figure 2 and Figure S2). This prediction was confirmed by the integrative analysis of ChIP-seq data of E-box binding proteins in the same cell line (Figure 2b and Figure S3). Of the E-box binding proteins, USF1 and USF2 were the most prominent E-box binding transcription factors, which coincided with RAD51. However, adequate experimental validation is needed to support this prediction. Second, we found that the promoters of a specific gene set were significantly occupied by RAD51 in four different cancer cell lines (Figure 1, Figure 2, and Figure S3). This means that there may be a conserved gene regulatory circuit in which RAD51 and E-box binding proteins are involved, and that activating this regulatory circuit is beneficial to cancer cells. This concept is partially supported by the fact that almost all cancer tissues showed an overexpression of RAD51, as compared to the corresponding normal tissues (Figure 4). Intriguingly, one of the predicted RAD51-mediated pathways turned out to be the autophagy pathway (Figure 3). This is the first report proposing a novel role of RAD51 as a potential transcriptional co-factor, capable of controlling genes that contribute to the autophagy pathway in cancer cells. The exact mechanism that RAD51 contributes is still unclear, but this unexpected finding underpins recent studies that there may be a mechanistic link between homologous recombination and autophagy, despite their occurrence in spatially distinct cellular compartments [21,44].

The paradoxical role of autophagy in cancer cells has been documented. For example, the role of autophagy in cancer initiation and epithelial–mesenchymal transition (EMT) is antitumoral, while its role in the growth of the primary tumor and anoikis resistance is protumoral [45]. Thus, the role of autophagy in cancer cells is context dependent. Based on the RAD51-mediated molecular mechanism predicted in this study, possibly linking homologous recombination and autophagy, we propose the following hypothesis that should be validated in the near future. Cancer cells generally proliferate abnormally and survive for longer than a normal cell’s life span [46]. In order for cancer cells to survive under stressful conditions caused by rapid proliferation, the autophagy pathway must be activated [47,48]. In addition, chromosome breaks that spontaneously occur in proliferating cells must be repaired through RAD51 [49]. Based on our results, we propose that RAD51 is involved in these two processes. In particular, our results suggest that RAD51 may modulate the expression of genes that are important for autophagy with E-box binding proteins such as USF1, USF2, and/or MITF, while acting on homologous recombination by filamenting on DNA single or double-stranded breaks for DNA repair and replication [50,51]. Nevertheless, the present study has the following limitations that need to be addressed in the near future. Although the identification of genome-wide RAD51 binding sites was based on quality-controlled RAD51 ChIP-seq experiments conducted by the ENCODE consortium, the motif and co-occupancy analyses were in silico analyses. In addition, the significant overlap of genomic binding sites between RAD51 and E-box binding proteins only provided evidence of co-localization, not direct interactions between those proteins. Furthermore, ChIP-seq data of autophagy and lysosome biogenesis-related transcription factors, such as transcription factor EB (TFEB), were not in the ENCODE database. Therefore, further work is required to prove the multiple roles of RAD51 in homologous recombination and autophagy to clarify whether they operate separately or in cooperation in the RAD51–autophagy axis.

## 5. Conclusions

We reanalyzed RAD51 ChIP-seq data collected from GM12878, HepG2, K562, and MCF-7 cell lines and identified 5137, 2611, 7192, and 3498 RAD51-associated *cis*-regulatory elements, the predicted functions of which were related to the autophagy pathway. Our integrative analysis of various transcription factor ChIP-seq data further showed that RAD51 may contribute to gene regulation through USF1 and USF2. Although results from our study provide the first in silico evidence of RAD51 contributing to the autophagy pathway, through RAD51-associated *cis*-regulatory elements, experimental validations are warranted to verify these findings.

## Data Availability

ChIP-seq data were downloaded from the ENCODE database (https://www.encodeproject.org/, accessed on 12 March 2021). The accession numbers for all samples can be found in Table S1.

## Supplementary Materials

The following are available online at https://www.mdpi.com/article/10.3390/cancers13112547/s1, Figure S1: Characteristics of RAD51 binding sites in enhancer and promoter regions, Figure S2: Motif analysis of H3K4me3-enriched regions, Figure S3: Comparative analysis of binding sites of RAD51- and E-box-related proteins, Figure S4: Expression levels of the RAD51 gene in various cancer cell lines, Figure S5. Knockdown of RAD51 in HepG2 and MCF-7 cells, Table S1: ChIP-seq data reanalyzed in this study, Table S2: Primers for RT-qPCR, Table S3: RAD51 binding sites identified in GM12878, HepG2, K562, or MCF-7 cells, Table S4: Common genes that were predicted to be regulated by RAD51 in all four cell lines.

## References

[1] Baumann P., West S.C. (1998). Role of the Human RAD51 Protein in Homologous Recombination and Double-Stranded-Break Repair. Trends Biochem. Sci..

[2] Bindra R.S., Schaffer P.J., Meng A., Woo J., Lizardi P., Hedley D.W., Bristow R.G., Glazer P.M. (2004). Down-Regulation of Rad51 and Decreased Homologous Recombination in Hypoxic Cancer Cells. Mol. Cell. Biol..

[3] Vispe S., Cazaux C., Lesca C., Defais M. (1998). Overexpression of Rad51 Protein Stimulates Homologous Recombination and Increases Resistance of Mammalian Cells to Ionizing Radiation. Nucleic Acids Res..

[4] Mazin A.V., Zaitseva E., Sung P., Kowalczykowski S.C. (2000). Tailed Duplex DNA Is the Preferred Substrate for Rad51 Protein-Mediated Homologous Pairing. EMBO J..

[5] Mason J.M., Chan Y.-L., Weichselbaum R.W., Bishop D.K. (2019). Non-Enzymatic Roles of Human RAD51 at Stalled Replication Forks. Nat. Commun..

[6] Bhattacharya S., Srinivasan K., Abdisalaam S., Su F., Raj P., Dozmorov I., Mishra R., Wakeland E.K., Ghose S., Mukherjee S. (2017). RAD51 Interconnects between DNA Replication, DNA Repair and Immunity. Nucleic Acids Res..

[7] Ko J.-C., Hong J.-H., Wang L.-H., Cheng C.-M., Ciou S.-C., Lin S.-T., Jheng M.-Y., Lin Y.-W. (2008). Role of Repair Protein Rad51 in Regulating the Response to Gefitinib in Human Non-Small Cell Lung Cancer Cells. Mol. Cancer Ther..

[8] Nagathihalli N.S., Nagaraju G. (2011). RAD51 as a Potential Biomarker and Therapeutic Target for Pancreatic Cancer. Biochim. Biophys. Acta.

[9] Hine C.M., Seluanov A., Gorbunova V. (2008). Use of the Rad51 Promoter for Targeted Anti-Cancer Therapy. Proc. Natl. Acad. Sci. USA.

[10] Makino E., Fröhlich L.M., Sinnberg T., Kosnopfel C., Sauer B., Garbe C., Schittek B. (2020). Targeting Rad51 as a Strategy for the Treatment of Melanoma Cells Resistant to MAPK Pathway Inhibition. Cell Death Dis..

[11] Tsai M.-S., Kuo Y.-H., Chiu Y.-F., Su Y.-C., Lin Y.-W. (2010). Down-Regulation of Rad51 Expression Overcomes Drug Resistance to Gemcitabine in Human Non–Small-Cell Lung Cancer Cells. J. Pharmacol. Exp. Ther..

[12] Qiao G.-B., Wu Y.-L., Yang X.-N., Zhong W.-Z., Xie D., Guan X.-Y., Fischer D., Kolberg H.-C., Kruger S., Stuerzbecher H.-W. (2005). High-Level Expression of Rad51 Is an Independent Prognostic Marker of Survival in Non-Small-Cell Lung Cancer Patients. Br. J. Cancer.

[13] Maacke H., Jost K., Opitz S., Miska S., Yuan Y., Hasselbach L., Lüttges J., Kalthoff H., Stürzbecher H.-W. (2000). DNA Repair and Recombination Factor Rad51 Is Over-Expressed in Human Pancreatic Adenocarcinoma. Oncogene.

[14] Maacke H., Opitz S., Jost K., Hamdorf W., Henning W., Ger S.K., Feller A.C., Lopens A., Diedrich K., Schwinger E. (2000). Over-Expression of Wild-Type Rad51 Correlates with Histological Grading of Invasive Ductal Breast Cancer. Int. J. Cancer.

[15] Chen J.-J., Silver D., Cantor S., Livingston D.M., Scully R. (1999). BRCA1, BRCA2, and Rad51 Operate in a Common DNA Damage Response Pathway. Cancer Res..

[16] Schild D., Wiese C. (2010). Overexpression of RAD51 Suppresses Recombination Defects: A Possible Mechanism to Reverse Genomic Instability. Nucleic Acids Res..

[17] Jia Y., Song Y., Dong G., Hao C., Zhao W., Li S., Tong Z. (2019). Aberrant Regulation of RAD51 Promotes Resistance of Neoadjuvant Endocrine Therapy in ER-Positive Breast Cancer. Sci. Rep..

[18] Kimmelman A.C. (2011). The Dynamic Nature of Autophagy in Cancer. Genes Dev..

[19] White E. (2012). Deconvoluting the Context-Dependent Role for Autophagy in Cancer. Nat. Rev. Cancer.

[20] Wang L., Howell M.E.A., Sparks-Wallace A., Hawkins C., Nicksic C.A., Kohne C., Hall K.H., Moorman J.P., Yao Z.Q., Ning S. (2019). P62-Mediated Selective Autophagy Endows Virus-Transformed Cells with Insusceptibility to DNA Damage under Oxidative Stress. PLoS Pathog..

[21] Hewitt G., Korolchuk V.I. (2017). Repair, Reuse, Recycle: The Expanding Role of Autophagy in Genome Maintenance. Trends Cell Biol..

[22] Zhu X., Pan Q., Huang N., Wu J., Zhen N., Sun F., Li Z., Yang Q. (2016). RAD51 Regulates CHK1 Stability via Autophagy to Promote Cell Growth in Esophageal Squamous Carcinoma Cells. Tumor Biol..

[23] Mo N., Lu Y.-K., Xie W.-M., Liu Y., Zhou W.-X., Wang H.-X., Nong L., Jia Y.-X., Tan A.-H., Chen Y. (2014). Inhibition of Autophagy Enhances the Radiosensitivity of Nasopharyngeal Carcinoma by Reducing Rad51 Expression. Oncol. Rep..

[24] Xie C., Li N., Wang H., He C., Hu Y., Peng C., Ouyang Y., Wang D., Xie Y., Chen J. (2020). Inhibition of Autophagy Aggravates DNA Damage Response and Gastric Tumorigenesis via Rad51 Ubiquitination in Response to *H. Pylori* Infection. Gut Microbes.

[25] Zai W., Chen W., Han Y., Wu Z., Fan J., Zhang X., Luan J., Tang S., Jin X., Fu X. (2020). Targeting PARP and Autophagy Evoked Synergistic Lethality in Hepatocellular Carcinoma. Carcinogenesis.

[26] Yang Q., Wu J., Luo Y., Huang N., Zhen N., Zhou Y., Sun F., Li Z., Pan Q., Li Y. (2016). (−)-Guaiol Regulates RAD51 Stability via Autophagy to Induce Cell Apoptosis in Non-Small Cell Lung Cancer. Oncotarget.

[27] Singh S.S., Vats S., Chia A.Y.-Q., Tan T.Z., Deng S., Ong M.S., Arfuso F., Yap C.T., Goh B.C., Sethi G. (2018). Dual Role of Autophagy in Hallmarks of Cancer. Oncogene.

[28] Aymard F., Bugler B., Schmidt C.K., Guillou E., Caron P., Briois S., Iacovoni J.S., Daburon V., Miller K.M., Jackson S.P. (2014). Transcriptionally Active Chromatin Recruits Homologous Recombination at DNA Double-Strand Breaks. Nat. Struct. Mol. Biol..

[29] Hazan I., Monin J., Bouwman B.A.M., Crosetto N., Aqeilan R.I. (2019). Activation of Oncogenic Super-Enhancers Is Coupled with DNA Repair by RAD51. Cell Rep..

[30] Davis C.A., Hitz B.C., Sloan C.A., Chan E.T., Davidson J.M., Gabdank I., Hilton J.A., Jain K., Baymuradov U.K., Narayanan A.K. (2018). The Encyclopedia of DNA Elements (ENCODE): Data Portal Update. Nucleic Acids Res..

[31] Langmead B., Salzberg S.L. (2012). Fast Gapped-Read Alignment with Bowtie 2. Nat. Methods.

[32] Tarasov A., Vilella A.J., Cuppen E., Nijman I.J., Prins P. (2015). Sambamba: Fast Processing of NGS Alignment Formats. Bioinformatics.

[33] Heinz S., Benner C., Spann N., Bertolino E., Lin Y.C., Laslo P., Cheng J.X., Murre C., Singh H., Glass C.K. (2010). Simple Combinations of Lineage-Determining Transcription Factors Prime Cis-Regulatory Elements Required for Macrophage and B Cell Identities. Mol. Cell.

[34] Quinlan A.R., Hall I.M. (2010). BEDTools: A Flexible Suite of Utilities for Comparing Genomic Features. Bioinformatics.

[35] Zhou Y., Zhou B., Pache L., Chang M., Khodabakhshi A.H., Tanaseichuk O., Benner C., Chanda S.K. (2019). Metascape Provides a Biologist-Oriented Resource for the Analysis of Systems-Level Datasets. Nat. Commun..

[36] Heberle H., Meirelles G.V., da Silva F.R., Telles G.P., Minghim R. (2015). InteractiVenn: A Web-Based Tool for the Analysis of Sets through Venn Diagrams. BMC Bioinform..

[37] Robinson J.T. (2011). Integrative Genomics Viewer. Nat. Biotechnol..

[38] Ramírez F., Dündar F., Diehl S., Grüning B.A., Manke T. (2014). DeepTools: A Flexible Platform for Exploring Deep-Sequencing Data. Nucleic Acids Res..

[39] Anaya J. (2016). OncoLnc: Linking TCGA Survival Data to MRNAs, MiRNAs, and LncRNAs. PeerJ Comput. Sci..

[40] Kang K., Choi Y., Kim H.H., Yoo K.H., Yu S. (2020). Predicting FOXM1-Mediated Gene Regulation through the Analysis of Genome-Wide FOXM1 Binding Sites in MCF-7, K562, SK-N-SH, GM12878 and ECC-1 Cell Lines. Int. J. Mol. Sci..

[41] Ristic D. (2005). Human Rad51 Filaments on Double- and Single-Stranded DNA: Correlating Regular and Irregular Forms with Recombination Function. Nucleic Acids Res..

[42] Xu J., Zhao L., Xu Y., Zhao W., Sung P., Wang H.-W. (2017). Cryo-EM Structures of Human RAD51 Recombinase Filaments during Catalysis of DNA-Strand Exchange. Nat. Struct. Mol. Biol..

[43] Qi Z., Redding S., Lee J.Y., Gibb B., Kwon Y., Niu H., Gaines W.A., Sung P., Greene E.C. (2015). DNA Sequence Alignment by Microhomology Sampling during Homologous Recombination. Cell.

[44] Gomes L., Menck C., Leandro G. (2017). Autophagy Roles in the Modulation of DNA Repair Pathways. Int. J. Mol. Sci..

[45] Dikic I., Elazar Z. (2018). Mechanism and Medical Implications of Mammalian Autophagy. Nat. Rev. Mol. Cell Biol..

[46] Feitelson M.A., Arzumanyan A., Kulathinal R.J., Blain S.W., Holcombe R.F., Mahajna J., Marino M., Martinez-Chantar M.L., Nawroth R., Sanchez-Garcia I. (2015). Sustained Proliferation in Cancer: Mechanisms and Novel Therapeutic Targets. Semin. Cancer Biol..

[47] White E. (2015). The Role for Autophagy in Cancer. J. Clin. Investig..

[48] Levy J.M.M., Towers C.G., Thorburn A. (2017). Targeting Autophagy in Cancer. Nat. Rev. Cancer.

[49] Sonoda E. (1998). Rad51-Deficient Vertebrate Cells Accumulate Chromosomal Breaks Prior to Cell Death. EMBO J..

[50] Kim T.M., Ko J.H., Hu L., Kim S.-A., Bishop A.J.R., Vijg J., Montagna C., Hasty P. (2012). RAD51 Mutants Cause Replication Defects and Chromosomal Instability. Mol. Cell. Biol..

[51] Yoon S.-W., Kim D.-K., Kim K.P., Park K.-S. (2014). Rad51 Regulates Cell Cycle Progression by Preserving G2/M Transition in Mouse Embryonic Stem Cells. Stem Cells Dev..

