# Supplementary material for: Epigenomic Analysis of RAD51 ChIP-seq Data Reveals cis-regulatory Elements Associated with Autophagy in Cancer Cell Lines
Source: Cancers (Basel). 2021 May 22;13(11):2547. doi: 10.3390/cancers13112547 (PMC8196894; doi:10.3390/cancers13112547)
Supplement: Supplementary file 1 [file cancers-13-02547-s001.zip › Supplementary_Figures.pdf]

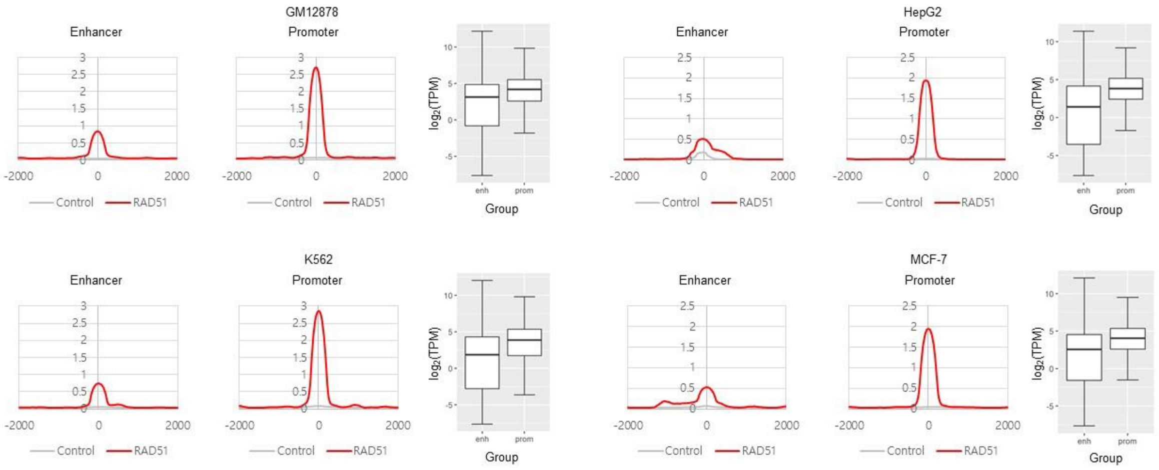

Figure S1. Characteristics of RAD51 binding sites in enhancer and promoter regions. Line plots show average enrichment of RAD51 binding at RAD51 binding sites in enhancer and promoter regions. Box plots show the expression level of genes closest to the RAD51 binding sites in enhancer and promoter regions.

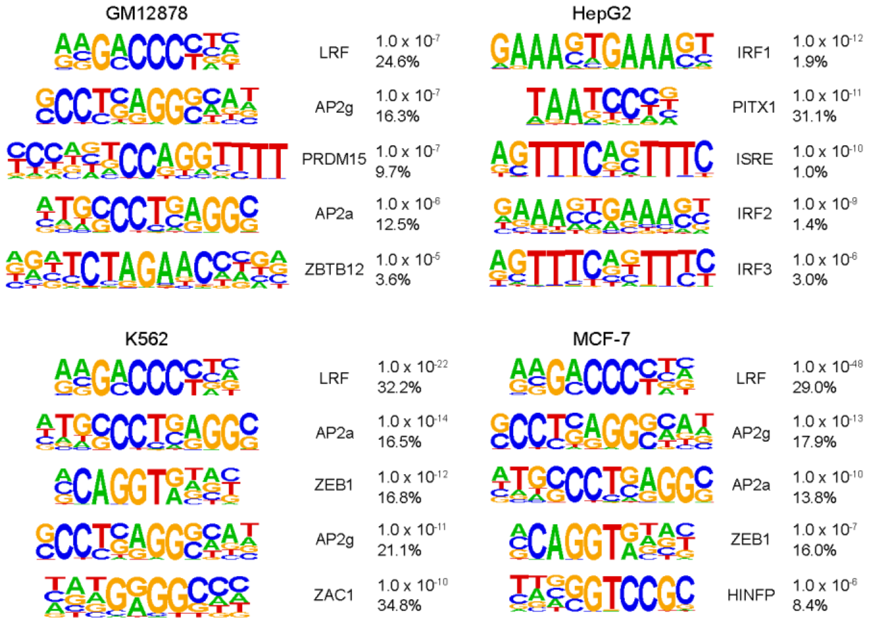

Figure S2. Motif analysis of H3K4me3-enriched regions. The top five motifs are shown.

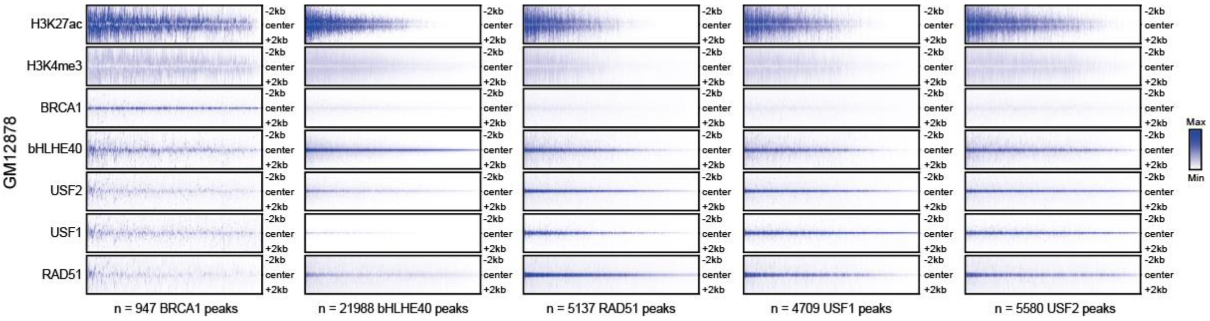

Figure S3. Comparative analysis of binding sites of RAD51- and E-box-related proteins. Heatmaps show enrichment of RAD51- and E-box-related proteins at the binding sites of a given protein (bottom) in GM12878 cells.

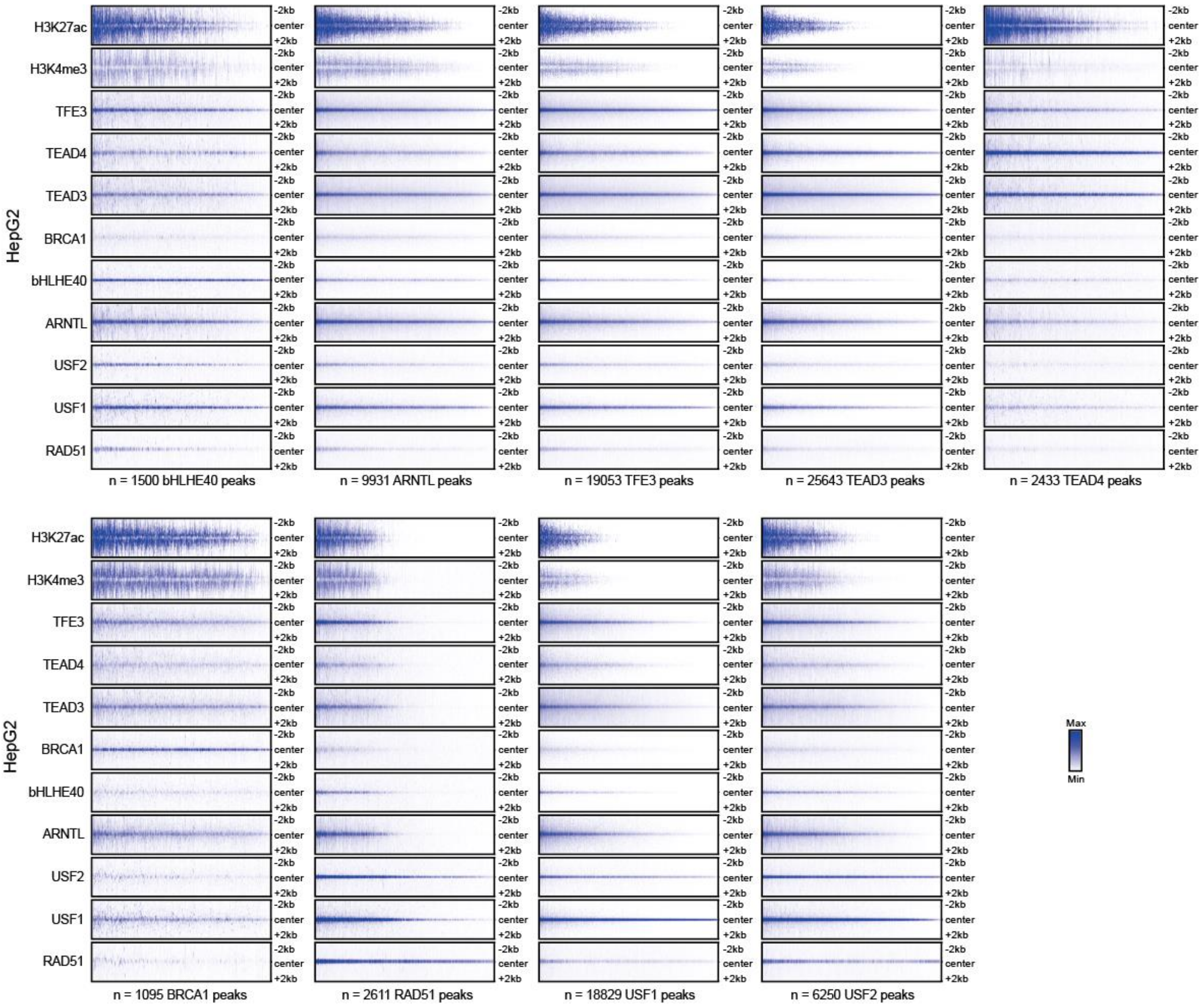

Figure S3 (continued). Comparative analysis of binding sites of RAD51- and E-box-related proteins. Heatmaps show enrichment of RAD51- and E-box-related proteins at the binding sites of a given protein (bottom) in HepG2 cells.

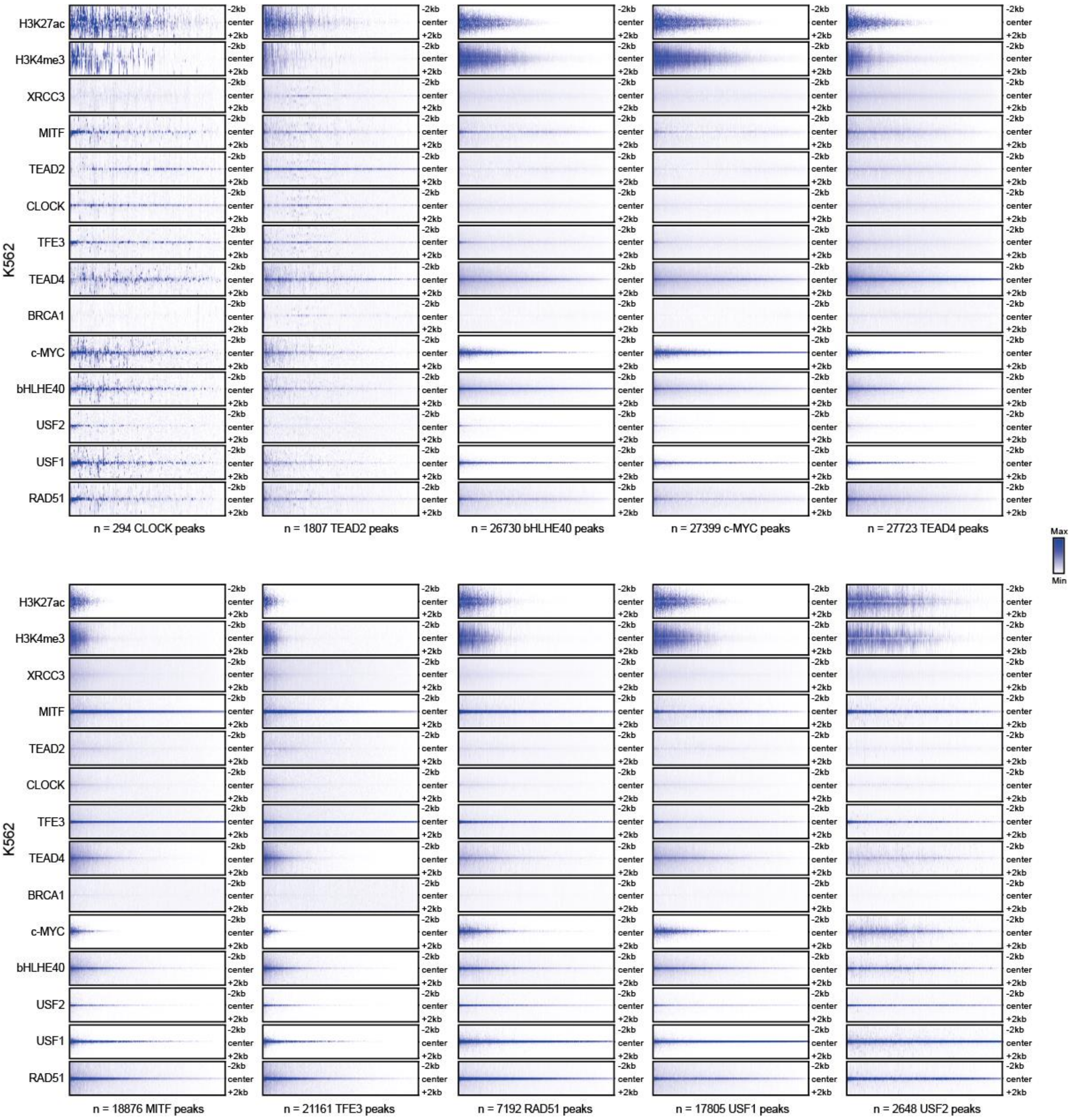

Figure S3 (continued). Comparative analysis of binding sites of RAD51- and E-box-related proteins. Heatmaps show enrichment of RAD51- and E-box-related proteins at the binding sites of a given protein (bottom) in K562 cells.

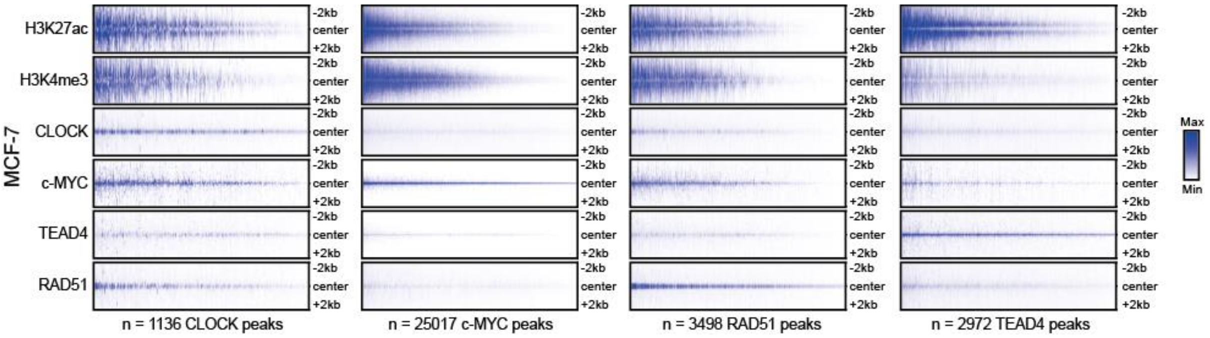

Figure S3 (continued). Comparative analysis of binding sites of RAD51- and E-box-related proteins. Heatmaps show enrichment of RAD51- and E-box-related proteins at the binding sites of a given protein (bottom) in MCF-7 cells.

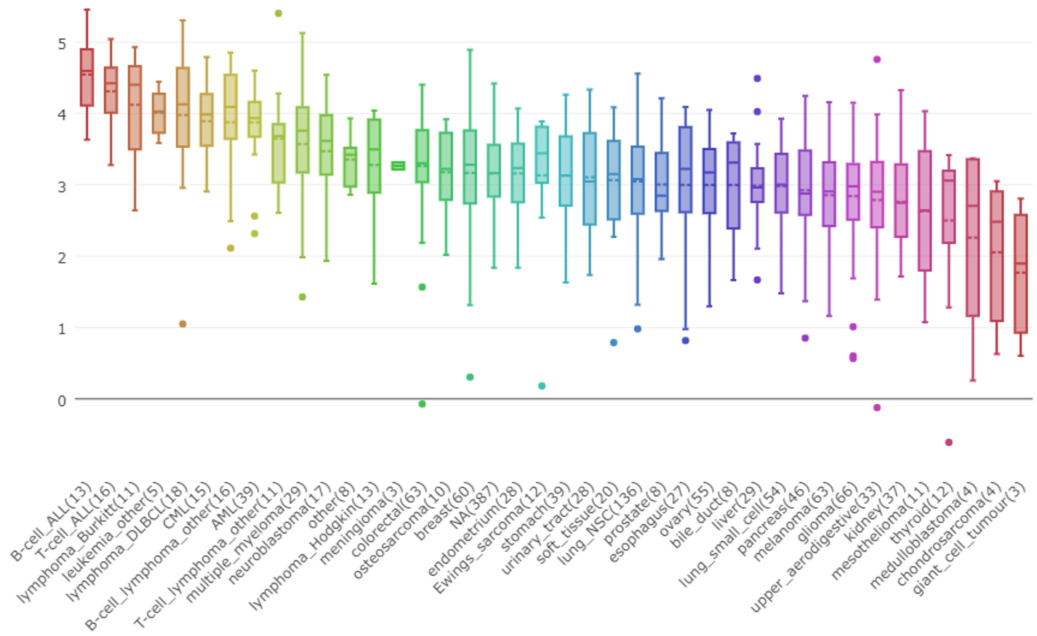

Figure S4. Expression levels of the *RAD51* gene in various cancer cell lines. The boxplot is colored by average distribution of the *RAD51* gene's expression in a lineage. Lineages are composed of cell lines (in parentheses; n = 1457 cancer cell lines) from the same area or system of the body. The data was obtained from the cancer cell line encyclopedia (CCLE) website (https://portals.broadinstitute.org/ccle).

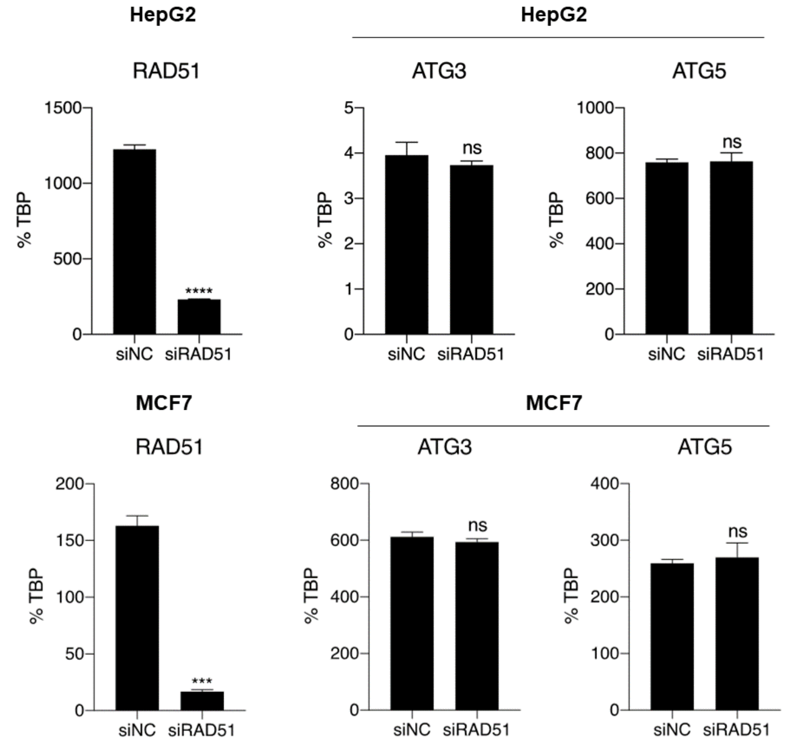

Figure S5. Knockdown of RAD51 in HepG2 and MCF-7 cells. HepG2 and MCF-7 cell lines were transfected with non-targeting (NC) siRNA or RAD51 siRNA. Real-time PCR analysis of RAD51 expression in HepG2 and MCF-7 cell lines showed the knockdown effect of RAD51 siRNA. HepG2 and MCF-7 cells were transfected with NC siRNA and RAD51 siRNA, and changes in the expression of ATG3 and ATG5, which are target genes of RAD51, were determined by real-time PCR analysis. *$p < 0.05$; **$p < 0.01$; ***$p < 0.001$; ****$p < 0.0001$. The analysis was performed using Welch's t-test. Data are shown as mean ± SEM.
